# The endocrine basis of the cardio‐renal axis: New perspectives regarding corin

**DOI:** 10.14814/phy2.16105

**Published:** 2024-06-28

**Authors:** Zaid Abassi, Dalit B. Hamo‐Giladi, Safa Kinaneh, Samuel N. Heyman

**Affiliations:** ^1^ Ruth & Bruce Rappaport Faculty of Medicine, Technion‐IIT Haifa Israel; ^2^ Department of Laboratory Medicine Rambam Health Care Campus Haifa Israel; ^3^ Department of Medicine Hadassah Hebrew University Hospital, Mt. Scopus and Herzog Hospital Jerusalem Israel

**Keywords:** cardiorenal axis, corin, edema, natriuresis, natriuretic peptides

## Abstract

The central role of natriuretic peptides (NPs) in the complex cardio‐renal integrated physiology and organ failure has been revealed over the last four decades. Atrial natriuretic peptide (ANP), the oldest representative of the NPs family, is produced through conversion of proANP to the mature peptide by corin, a trans‐membrane protease localized to the cardiac myocyte membrane. Similarly, brain natriuretic peptide (BNP) is generated by furin, which cleaves proBNP to BNP in myocytes. Though the components of NPs system, their synthesis and target organs are well established, understanding their role in the interplay between the heart and the kidney is steadily evolving. In this context, Feldman et al. (New England Journal of Medicine, 389, 1685) recently described patients with hypertension, cardiomyopathy, atrial arrhythmia and left atrial fibrosis, associated with a homozygous loss‐of‐function variant of the gene encoding corin (Cor^−/−^). Notably, reduced baseline urinary electrolyte and creatinine excretion have been observed in one of the studied patients. This renal excretory functional impairment could be attributed to the lack of cardiac‐derived ANP in these patients, as implied by Feldman et al. Yet, in this mini‐review we suggest that this aberrant renal manifestation may principally stem from lack of local ANP production at renal tissue, as corin is normally expressed in proximal tubules, Henle's loop and collecting ducts, with locally produced ANP provoking Na^+^ and water exertion. Collectively, it seems that beside the classic well‐established cardio‐renal axis, the renal NPs system functions as local endocrine machinery in the regulation of sodium excretion.

Since the discovery of atrial natriuretic peptide (ANP) four decades ago (Flynn et al., 1983), major developments have taken place in our understanding of the role of the heart as an endocrine organ, under both physiological and pathophysiological conditions. ANP, a 28 aa peptide, represents a broader family of natriuretic peptides (NPs) consisted of at least two additional members, namely brain natriuretic peptide (BNP) and C‐peptide natriuretic peptide (CNP), encoded by different genes: NPPA, NPPB and NPPC, respectively (Goetze et al., 2020). While ANP is released mainly from the right atrium either constitutively or in response to atrial destination due to volume expansion, BNP is secreted from the cardiac ventricles in response to volume or pressure overload (Goetze et al., 2020) (Figure 1a). The biological actions of both ANP and BNP are mediated via a specific membranal receptor, NPR‐A coupled with the activation of an intracellular signal transduction system using guanosine 3′,5′‐cyclic monophosphate (cGMP) as a second messenger (Goetze et al., 2020). In contrast, CNP, which shares structural similarities with ANP and BNP, lacks a natriuretic effect as it acts via NPR‐B localized to bone, fibroblasts and the uterus, suggesting a different biological role for the CNP–NPR‐ B system from that of the cardiac NPs (Goetze et al., 2020). The involvement of NPs in maintaining body fluid and electrolyte homeostasis and the regulation of blood pressure is well established. In this context, circulating ANP and BNP have powerful natriuretic activity (Goetze et al., 2020; McGregor et al., 1990) along vasorelaxant properties due to relaxation of vascular smooth muscle and mesangial cells (Levin et al., 1998), suppression of the renin angiotensin aldosterone system and the sympathetic nervous system, and acts on the central nervous system to modulate vasomotor tone, thirst, and vasopressin release (Goetze et al., 2020; Song et al., 2015). Furthermore, NPs also exert anti‐proliferative and anti‐fibrotic activity in cardiac tissue, glomeruli and at the vasculature, effects that may constitute the basis for their cardiac and renal protective properties (Goetze et al., 2020; Song et al., 2015). NPs are produced from pro‐NPs, where ANP and BNP are generated by the conversion of pro‐ANP and pro‐BNP into ANP and BNP, executed by corin and furin, respectively (Semenov et al., 2010) (Figure 1a). While corin is a membrane‐bound serine protease, generating ANP, furin is a cytosolic and membranal enzyme responsible for the activation of BNP (Goetze et al., 2020; Song et al., 2015). Although, the heart is considered as a major site of ANP and BNP synthesis, numerous studies have explicitly demonstrated that some or all the components of NPs system are also expressed in non‐cardiac tissues including the lung and kidney (Abassi et al., 2022; Khoury et al., 2018; Khoury, Fokra, et al., 2021; Khoury, Knaney, et al., 2021). Renal ANP is produced in most segments of renal tubules (Dong et al., 2016). Moreover, BNP is also expressed in different tubule segments (Beltowski & Wojcicka, 2002), as immunocytochemistry revealed that immunoreactive BNP was colocalized with immunoreactive ANP in the segments of distal tubules (Totsune et al., 1994). ANP/BNP bind to NPR‐A coupled to intracellular cGMP production, and eventually provokes natriuresis and diuresis (Goetze et al., 2020; Song et al., 2015). The latter are mainly mediated by the inhibitory action of ANP on the basolaterally expressed Na^+^‐K^+^‐ATPase, reducing apical sodium, potassium, and protein organic cation transporter in the proximal tubule (PT), and through decreasing Na^+^‐K^+^‐2Cl^−^ cotransporter activity and renal concentration efficiency in the thick ascending limb (TAL). In medullary collecting ducts (CD), ANP reduces sodium reabsorption by inhibiting the epithelial sodium channel (ENaC) (Theilig & Wu, 2015). Interestingly, analysis of renal expression of corin in human and rats revealed that it co‐localized with ANP in epithelial cells, with expression in the PT, TAL and throughout the CD (Dong et al., 2016; Polzin et al., 2010). Specifically, Dong et al. (Dong et al., 2016) reported that corin expression was most abundant in the proximal convoluted tubules and the medullary connecting ducts of human kidney, and to a lesser extent in the TAL. Similar distribution pattern of renal ANP/NRP‐A expression has been noted. Noteworthy, these authors found a defuse pattern of pro‐ANP/ANP staining in the cytoplasm of the proximal tubular epithelial cells. Similarly, Polzin et al. (Polzin et al., 2010) reveal that renal corin immunostaining was found in epithelial cells, with segmental expression in the PT, TAL, connecting tubule (CNT), and throughout the CD, with the highest expression taking place in the medulla. In line with Dong et al. (Dong et al., 2016) findings, corin immunohistochemistry co‐localized with ANP. Given the similar corin, pro‐ANP/ANP and NPR‐A expression patterns in the same renal segment, corin‐generated ANP is likely to act in situ in the proximal convoluted tubules and probably in other segments of the tubule (Dong et al., 2016; Polzin et al., 2010).

**FIGURE 1:**
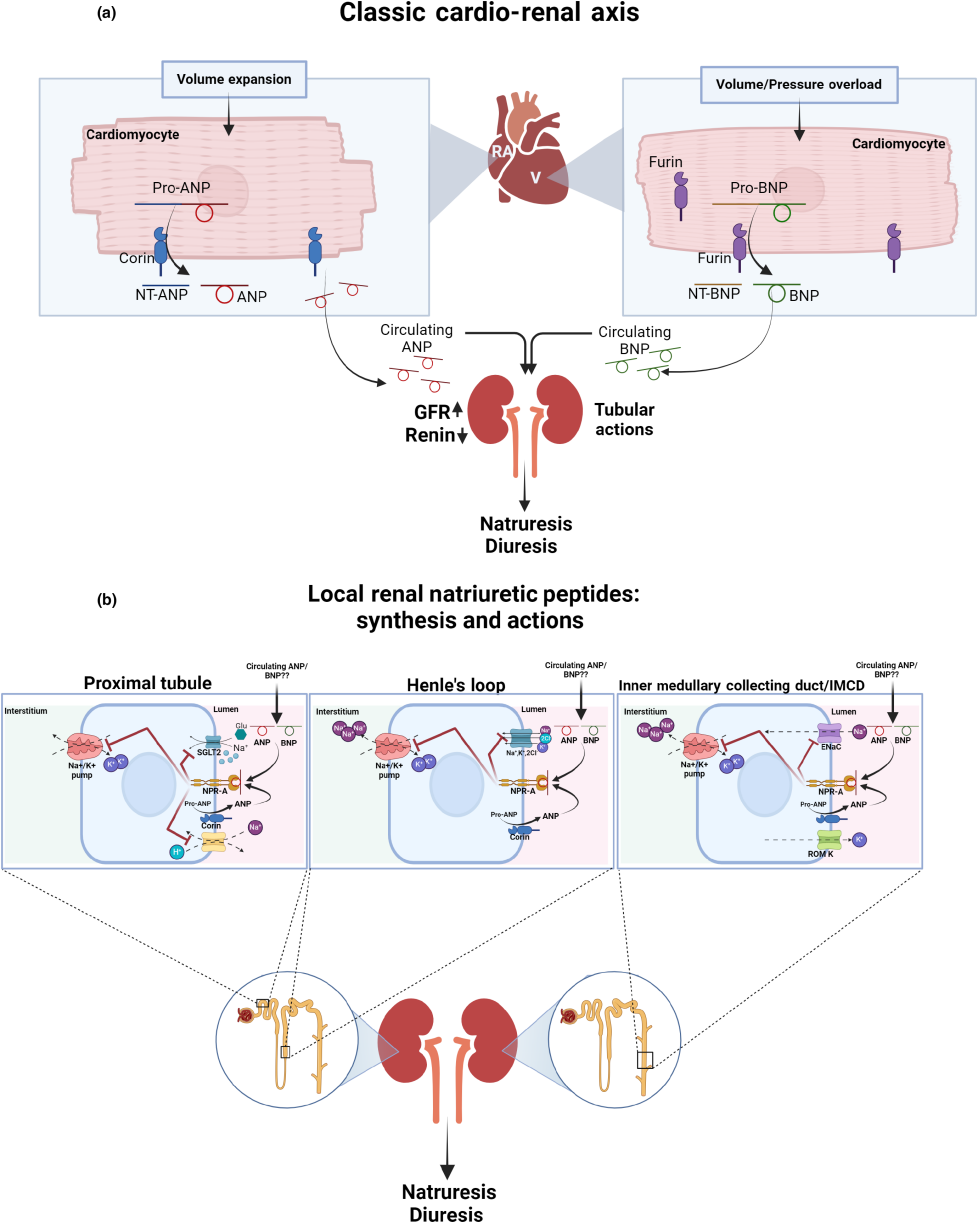
(a) Classic cardio‐renal axis: Volume/sodium homeostasis is controlled by natriuretic peptides (NPs), namely atrial natriuretic peptide (ANP) secreted in response to right atrium distention and brain natriuretic peptide (BNP) secreted from cardiac ventricles in response to volume/pressure overload. While, ANP is produced through conversion of proANP to the mature peptide by corin, a trans‐membrane protease localized to the cardiac myocyte membrane, brain natriuretic peptide (BNP) is generated by furin, which cleaves proBNP to BNP in myocytes. Both ANP and BNP acts via NPR‐A in the kidney to increase urine output and Na^+^ excretion, thus reducing extracellular volume. These effects are due to NPs actions on the glomerulus, where they increase glomerular perfusion and filtration, and the tubule, where they inhibit Na^+^ reabsorption at proximal tubules, Henle's loop and collecting duct (CD). (b) Local Renal Natriuretic Peptides‐Synthesis and Actions: Besides the classic cardio‐renal axis described above, the kidney contains in situ components of NPs system, namely corin, ANP and NPR‐A, that act in auto/paracrine manner to enhance Na^+^ and H_2_O excretion. By converting NPs to their active form, corin constitutes an essential player in the regulation of water and salt balance in both physiological and pathophysiological conditions. Specifically, corin is expressed in the proximal tubule, thick ascending Henle's loop (TAL) and collecting duct, co‐localized with ANP and NPR‐A. Thus, it is tempting to assume that locally produced ANP may act in situ in the kidney to regulate Na^+^ and water balance. Collectively, these findings suggest that renal NPs functions as local endocrine system with certain degree of independency from the well‐established classic cardiac NPs system, which plays a keen role in cardio‐renal axis. ANP, atrial natriuretic peptide; BNP, brain natriuretic peptide; Cl^−^, chloride; IMCD, inner collecting duct; ENaC, epithelial sodium channel; Na^+^, sodium; K^+^, potassium; NPR‐A, natriuretic peptide receptor type A; SGLT2, sodium glucose cotransporter.

Of notice, corin‐deficient (Cor^−/−^) mice express high levels of pro‐ANP with no detectable levels of ANP, and develop hypertension, exacerbated by salt load and pregnancy as do ANP‐ and guanylyl cyclase‐A‐ deficient mice (Chan et al., 2005). Interestingly, corin^−/−^ mice show increased levels of renal β‐ENaC and phosphodiesterase 5 (PDE5), possibly responsible for impaired renal excretory functions in these animals (Polzin et al., 2010). Edematous disease states are characterized by ANP resistance and corin deficiency, highlighting the importance of renal ANP signaling in salt and water balance and in kidney pathophysiology (Abassi et al., 2022).

Since most of the reported studies utilized either experimental models or clinical loss‐of‐function mutations of various components of the NPs system, including NPR‐A, ANP, and Cor^−/−^, affecting both the kidney and cardiac tissues, it is impossible to distinguish between the contribution of local alterations in either cardiac‐ or renal generation of NPs to the observed renal physiology.

In a pioneer study by Feldman et al. (Baris Feldman et al., 2023) two siblings are described with hypertension, cardiomyopathy, atrial arrythmia and left atrial fibrosis, associated with a homozygous loss‐of‐function variant of the gene encoding corin (Cor^−/−^). A plasma sample obtained from one of the siblings had no detectable levels of corin or N‐terminal pro‐ANP but had showed elevated levels of BNP. Furthermore, one of the studied patients displayed reduced baseline urinary electrolyte and creatinine excretion despite the high levels of circulating BNP, an agonist of NPR‐A. The authors suggested that BNP cannot fully compensate for a lack of activation of the ANP pathway and that corin is critical to normal ANP activity and for left atrial function and cardiovascular homeostasis. Yet this hypothesis has one major drawback: although variants in genes encoding ANP receptor‐NPR‐A have been linked to cardiovascular diseases (Chan et al., 2005; Goetze et al., 2020; Song et al., 2015), NPR‐A shows high affinity to both ANP and BNP. Thus, impaired NPR‐A activation could not solely explain the observed phenomenon, as a likely compensatory elevated BNP could activate this receptor subtype. Therefore, we would like to suggest an additional hypothesis where we assume that cardiac and renal NPs plays distinct independent roles beside the classic interplay between the heart and the kidney as described above (Figure 1a). In this context, the observed impaired kidney function in the studied patient could be attributed to low levels of plasma ANP in these patients as suggested by the authors; however, aberrant local renal corin/ANP/NPR‐A machinery could take place as well. This notion is supported by the fact that corin, is also expressed, as stated above, at the apical membrane of proximal tubules epithelial cells, Henle's loop and CD (Dong et al., 2016; Polzin et al., 2010) (Figure 1b), with locally produced ANP provoking Na^+^ and water exertion (Song et al., 2015; Theilig & Wu, 2015). Furthermore, keen back up for this hypothesis was derived from a genetically modified mice model, where corin conditional knockout (cKO), either in the kidney (kcKO) or heart (hcKO) were selectively disrupted (Zhou et al., 2022). When placed on a normal‐salt diet (0.3% NaCl), corin kcKO and hcKO mice had increased blood pressure, indicating that both renal and cardiac corin is necessary for normal blood pressure in mice. However, on a high‐salt diet (4% NaCl), corin kcKO mice, but not corin hcKO mice showed reduced urinary Na^+^ and Cl^−^ excretion, increased body weight, salt‐exacerbated hypertension, and cardiac hypertrophy. These findings indicate that renal corin plays an important role in enhancing natriuresis upon high salt intake and that this function may not be compensated by the cardiac‐generated corin (Figure 1a). This may underscore the plausible important apocrinic corin‐ANP association at the renal tubular level.

In summary, although the fundamental concept concerning the components of NPs system and its cardiac origin as well as its target organs are still valid, the last decade witnessed breakthrough in our understanding of this system. These new insights were derived mainly from either genetically modified animals or clinical loss‐of‐function mutations in the components of NPs system, where it is obvious today that the heart and kidney maintain keen interplay, but still in term of sodium balance, renal NPs functions as local endocrine system with a certain degree of independency.

## FUNDING INFORMATION

The authors are grateful for the Israeli Science Foundation (Grant # 544/18), and Dr. Y. Rabinovitz Research Fund‐Technion for their financial support (86160798).

## CONFLICT OF INTEREST STATEMENT

All authors have no conflict of interests in this article.

## ETHICS STATEMENT

None.

## Data Availability

Data available on request from the authors.

## References

[phy216105-bib-0001] Abassi, Z. , Khoury, E. E. , Karram, T. , & Aronson, D. (2022). Edema formation in congestive heart failure and the underlying mechanisms. Frontiers in Cardiovascular Medicine, 9, 933215. 10.3389/fcvm.2022.933215 36237903 PMC9553007

[phy216105-bib-0002] Baris Feldman, H. , Chai Gadot, C. , Zahler, D. , Mory, A. , Aviram, G. , Elhanan, E. , Shefer, G. , Goldiner, I. , Amir, Y. , Kurolap, A. , & Ablin, J. N. (2023). Corin and Left atrial cardiomyopathy, hypertension, arrhythmia, and fibrosis. The New England Journal of Medicine, 389, 1685–1692. 10.1056/NEJMoa2301908 37913506

[phy216105-bib-0003] Beltowski, J. , & Wojcicka, G. (2002). Regulation of renal tubular sodium transport by cardiac natriuretic peptides: Two decades of research. Medical Science Monitor, 8, RA39–RA52. 11859295

[phy216105-bib-0004] Chan, J. C. , Knudson, O. , Wu, F. , Morser, J. , Dole, W. P. , & Wu, Q. (2005). Hypertension in mice lacking the proatrial natriuretic peptide convertase corin. Proceedings of the National Academy of Sciences of the United States of America, 102, 785–790. 10.1073/pnas.0407234102 15637153 PMC545541

[phy216105-bib-0005] Dong, L. , Wang, H. , Dong, N. , Zhang, C. , Xue, B. , & Wu, Q. (2016). Localization of corin and atrial natriuretic peptide expression in human renal segments. Clinical Science, 130, 1655–1664. 10.1042/CS20160398 27343265 PMC5237585

[phy216105-bib-0006] Flynn, T. G. , de Bold, M. L. , & de Bold, A. J. (1983). The amino acid sequence of an atrial peptide with potent diuretic and natriuretic properties. Biochemical and Biophysical Research Communications, 117, 859–865. 10.1016/0006-291x(83)91675-3 6230081

[phy216105-bib-0007] Goetze, J. P. , Bruneau, B. G. , Ramos, H. R. , Ogawa, T. , de Bold, M. K. , & de Bold, A. J. (2020). Cardiac natriuretic peptides. Nature Reviews Cardiology, 17, 698–717. 10.1038/s41569-020-0381-0 32444692

[phy216105-bib-0008] Khoury, E. E. , Fokra, A. , Kinaneh, S. , Knaney, Y. , Aronson, D. , & Abassi, Z. (2021). Distribution of cardiac and renal corin and proprotein convertase subtilisin/kexin‐6 in the experimental model of cardio‐renal syndrome of various severities. Frontiers in Physiology, 12, 673497. 10.3389/fphys.2021.673497 34733169 PMC8558519

[phy216105-bib-0009] Khoury, E. E. , Kinaneh, S. , Aronson, D. , Amir, O. , Ghanim, D. , Volinsky, N. , Azzam, Z. , & Abassi, Z. (2018). Natriuretic peptides system in the pulmonary tissue of rats with heart failure: Potential involvement in lung edema and inflammation. Oncotarget, 9, 21715–21730. 10.18632/oncotarget.24922 29774097 PMC5955134

[phy216105-bib-0010] Khoury, E. E. , Knaney, Y. , Fokra, A. , Kinaneh, S. , Azzam, Z. , Heyman, S. N. , & Abassi, Z. (2021). Pulmonary, cardiac and renal distribution of ACE2, furin, TMPRSS2 and ADAM17 in rats with heart failure: Potential implication for COVID‐19 disease. Journal of Cellular and Molecular Medicine, 25, 3840–3855. 10.1111/jcmm.16310 33660945 PMC8014258

[phy216105-bib-0011] Levin, E. R. , Gardner, D. G. , & Samson, W. K. (1998). Natriuretic peptides. The New England Journal of Medicine, 339, 321–328. 10.1056/NEJM199807303390507 9682046

[phy216105-bib-0012] McGregor, A. , Richards, M. , Espiner, E. , Yandle, T. , & Ikram, H. (1990). Brain natriuretic peptide administered to man: Actions and metabolism. The Journal of Clinical Endocrinology and Metabolism, 70, 1103–1107. 10.1210/jcem-70-4-1103 2156886

[phy216105-bib-0013] Polzin, D. , Kaminski, H. J. , Kastner, C. , Wang, W. , Krämer, S. , Gambaryan, S. , Russwurm, M. , Peters, H. , Wu, Q. , Vandewalle, A. , Bachmann, S. , & Theilig, F. (2010). Decreased renal corin expression contributes to sodium retention in proteinuric kidney diseases. Kidney International, 78, 650–659. 10.1038/ki.2010.197 20613715 PMC4433746

[phy216105-bib-0014] Semenov, A. G. , Tamm, N. N. , Seferian, K. R. , Postnikov, A. B. , Karpova, N. S. , Serebryanaya, D. V. , Koshkina, E. V. , Krasnoselsky, M. I. , & Katrukha, A. G. (2010). Processing of pro‐B‐type natriuretic peptide: Furin and corin as candidate convertases. Clinical Chemistry, 56, 1166–1176. 10.1373/clinchem.2010.143883 20489134

[phy216105-bib-0015] Song, W. , Wang, H. , & Wu, Q. (2015). Atrial natriuretic peptide in cardiovascular biology and disease (NPPA). Gene, 569, 1–6. 10.1016/j.gene.2015.06.029 26074089 PMC4496260

[phy216105-bib-0016] Theilig, F. , & Wu, Q. (2015). ANP‐induced signaling cascade and its implications in renal pathophysiology. American Journal of Physiology. Renal Physiology, 308, F1047–F1055. 10.1152/ajprenal.00164.2014 25651559 PMC4436998

[phy216105-bib-0017] Totsune, K. , Takahashi, K. , Murakami, O. , Satoh, F. , Sone, M. , Saito, T. , Sasano, H. , Mouri, T. , & Abe, K. (1994). Natriuretic peptides in the human kidney. Hypertension, 24, 758–762. 10.1161/01.hyp.24.6.758 7995634

[phy216105-bib-0018] Zhou, T. T. , Zhang, S. , Du, C. , Wang, K. , Gu, X. , Sun, S. , Zhang, X. , Niu, Y. , Wang, C. , Liu, M. , Dong, N. , & Wu, Q. (2022). Renal Corin is essential for normal blood pressure and sodium homeostasis. International Journal of Molecular Sciences, 23, 11251. 10.3390/Ijms231911251 36232551 PMC9570390

